# Robert Jack and Louis Du Pasquier: Evolutionary Concepts in Immunology: 1st ed. (June 14, 2019); Springer Nature Switzerland AG; 145 pages (hardcover); ISBN 978-3-030-18666-1; ISBN 978-3-030-18667-8 (eBook), https://doi.org/10.1007/978-3-030-18667-8

**DOI:** 10.3325/cmj.2019.60.483

**Published:** 2019-10

**Authors:** Danka Grčević

**Field of medicine:** Immunology, biology.

**Format:** Hardcover book/eBook.

**Audience:** Immunology is a rapidly developing field, of great interest for researchers, clinicians, and students in a wide variety of biomedical and clinical areas including biology, hematology, infectious diseases, etc. The book tackles immunology from the evolutionary perspective in order to understand “forces that shaped, and are shaping, immune defense systems.” To fully appreciate the content, the reader would benefit from having basic knowledge of both fields.

**Purpose:** As stressed by the authors, the book does not attempt to comprehensively cover evolution or immunology, but rather “to present certain immune phenomena in an evolutionary context.” It certainly contributes to the book’s originality that the authors do not hesitate to give their personal interpretation of the presented information, Therefore, instead of being a dull collection of facts, the book aims to “provide a starting point for those who wish to find out more about particular issues.”

**Content:** The book is divided into six chapters: “What Makes Evolution Tick?,” “From Unicellular to Metazoan Immunity,” “Innate Immunity,” “The Triumph of Individualism: Evolution of Somatically Generated Adaptive Immune Systems,” “The Other Side of the Arms Race,” and “Postface,” followed by the Appendices and Index.

The first chapter gives a short overview of the fundamental evolutionary processes that would be further discussed in the context of immunology, such as heredity, mutations, genetic drift, recombination, etc. Within this chapter, the authors refer to postulates by Thomas Malthus, Charles Darwin, Herbert Spencer, Ernst Mayr, and some others, that are fundamental to the understanding of evolution. The chapter ends with the subheading “Evolution of Immunity,” referring to the specific evolutionary mechanisms within the immune system. In this chapter, the authors persuasively raise several intriguing points that make the reader curious to read the rest of the book.

The following few chapters focus on immunity, starting from the second chapter, which deals with the development of the immune system from unicellular to metazoan organisms. The concepts of lineage restriction, recognition of self, and mobilization of the immune machinery are presented together with examples, making the text easy to follow despite the relative sparsity of the graphical presentations. At the end of the chapter, authors discuss the important concept of immune response polarization into the innate and adaptive arm, referring to the “seminal remark made by Charles Janeway” describing the two systems as “dramatically different” but, at the same time, tightly integrated to achieve the common goal.

The third chapter deals with the innate immunity, explaining first the organization of the innate immune system and then the evolutionary processes, mainly focusing on the innate immune receptors. Several subheadings explain the mechanisms of gene duplication, modification, and exon/intron swapping, which, during evolution, have produced diversified intracellular and extracellular receptors capable of recognizing a potential threat. In addition to pathogen recognition, innate immune system is able to transmit a signal initiated by receptors and produce a “means of destruction” based on phagocytosis, effective even in “the single cell organisms that existed prior to the evolution of metazoans.”

The fourth chapter introduces adaptive immunity, explaining why we need more than the innate immunity to be able to recognize the antigen and produce a protective response. From the evolutionary perspective, “crucial difference is that innate systems are always germline based, while adaptive systems are always based on somatic adaptations.” Important mechanisms of such “somatic evolution of the immune system” are described related to immune receptors, formation of repertoire, ligand binding, etc. An important observation made by the authors is that evolution has suddenly changed “course,” resulting in some “exceptional events in the evolution of the antigen-specific receptors of adaptive immunity” in agnathans and gnathostomes.

The fifth chapter depicts the “battle” between the immune system and pathogens. After we arm ourselves with innate and adaptive “weapons,” we should test the competence by facing the enemy. But, evolution is a tricky force; “any effective defensive move made by the host merely provides the selection pressure that drives the evolution of new virulence strategies in pathogens,” resulting in a real “arms race.” In other words, pathogens are not helpless at all. During evolution, they have developed different strategies directed against the innate and adaptive immune system, named by the authors as “disrupting detection hardware,” “playing dead,” or “hiding in the immunological future.”

The Postface contains concluding remarks and the summary of authors’ opinions on the immune evolution. An important highlight is that “the evolution of immunity follows the principle of permanent revolution,” so nothing stays “quite the same.” The immune system is constantly changing in order to keep track with the evolution of pathogen virulence mechanisms. In this perspective, our “current understanding of the subject is no more than a mere snapshot of the present,” which means a lot of work for future generations!

**Highlights:** The book is rather concise, striving for originality at the expense of quantity. The language is not typically scientific; the book provides an original perspective in an unconventional and interesting manner. The authors give examples of the discussed phenomena, making it easier to follow the arguments. They also combine the citations of the seminal works in the field of evolution and immunology with scientific facts and personal opinions, producing a very intriguing text. As stressed by the authors, “immunity’s seemingly simple primary role of providing us with defense against pathogens is hedged about with innumerable ifs and buts.” So, the role of the book is to inspire us and motivate us to learn more.

**Related reading:** At the end of each chapter, the authors provide a list of references related to the text, as well as a list for further reading, which help guide further study and provide wider perspectives for the readers.

